# Dentistry in Brazil, South America

**Published:** 1871-08

**Authors:** W. F. Morrill


### DENTISTRY IN BRAZIL, SOUTH AMERICA.

BY W. F. MORRILL.

Read before the Indiana State Dental Society.

Gentlemen of the Indiana State Dental Association:—To-day, my absence from your meeting occasions me sincere regret. The social amenities and the scientific purposes are so happily blended in our gatherings, that not to be there, is seemingly to appear discourteous. Moreover, I am deprived of professional information—a matter of some moment to me. This absence will be, as if a leaf had been torn from a book—an interregnum in professional acquisition. And while I am not do-day permitted to share with you in the discussion of those topics pertinent to the welfare of dentistry, I trust another year I may be privileged to mingle with you where I have so many cherished remembrances. I herewith submit to your consideration observations connected with dental practice in Brazil.

It is known to some of you at least, that a disturbed condition of health made it necessary I should relinquish practice for a while, and repair to some more genial clime where the hygienic restoratives are supposed to reside. Brazil became the point of my destination. I reached the capital of this Empire on March 2nd, 1871, where I have since remained. The change derived from the effects of a sea-voyage, gave me my wonted vigor, and I soon after my arrival, entered upon dental practice. The city of Rio de Janeiro, with a population of 400,000, has more than 60 dentists. There are four American offices, while all others are either French or Brazilian. Two of these American offices would make a respectable standing among the offices of the United States. By this I mean to say the character of the operations done at these places will compare and be classed among the first in our country. With the occupants of the other two offices, I have had frequent opportunities to converse, and they each discouraged good operations, as the demand is for cheap and inexpensive kinds. As a N. Y. "Blood" in one of them told me he was a "slinger of amalgam," so I am permitted to adjudge his operations.

To open an office in Brazil for dental practice, where a sign can hang at the entrance on which is painted "Cirurgŭo dentistŭ;" a title from the Medical Faculty must be first secured. The applicant submits to a Medical Board for examination. Not one of these men comprising the Board comprehend a single principle of dentistry. Probably this Board might, if it was so disposed, conduct a *medical* examination of an applicant, but anything further than this, a like number of shoemakers would be equally as competent to decide upon the skill requisite to perform dental operations. Naturally enough, then, the law is a nullity. Brazil has its full quoto of dental quacks. A few paltry dollars placed in the hands of certain knowing ones, brings a "title" to the field of dentistry. Under the official seal of the Empire, a copy of the "title" runs thus:

"Empire of Brazil."

The Faculty of Medicine of Rio de Janeiro considering that Mr. —— ———, is a native of North America and the State of New Jersey, and a son of Mr. and Mrs. —— ———, was examined in dental art, ordered the passing of this title with which he will enjoy all the prerogatives the laws of this Empire, authorize those of his profession."

Subscribed by Medical Faculty and Secretary of the same.

The candidate thus panoplied can assail the public for dental patronage. Dental signs with letters of unmistakable size, stretching the entire length of the house, informing the public that the occupant is "dentist to the Imperial family," frequently greet the eye. If the Imperial family employ all of these men thus termed, then it is a most magnificent patron of dentistry.

Brazilians are usually blessed or cursed with lengthy names, and when "Cirurgũon Dentistu" is added, the whole is startling. But when "Barbeiro" is super-added, as is sometimes the case, it becomes fairly frightful. And whether these two professions, so amicably twinned in Brazil, require an additional "title," other than the one already given as under the Imperial sanction is included, I am not informed. Quite likely a Board sits specially to grant licenses to barbers! College diplomas from the States have no weight toward influencing a title-grant.

A young man from the States of some promise in his dental qualifications, made application for a title to practice, was very unfairly denied by this Board. He went home, hung out his shingle with merely his name, and to-day commands a fine practice. The people know the value of his operations, and his prices are above any other operator in Rio. By law without this title he can not collect money; but he chooses a better way, and collects his money as the work each day progresses. At the close of the day he knows no bills are to be collected.

My acquaintance with Brazilian and French dentists in this city has been limited, but quite enough to form a just opinion of the nature and character of their operations, and also to form some judgment of their scientific attainments. The performances on the teeth are universally bad. Amalgam forms the chief material and in many offices the *only* material for filling teeth. You might as well talk to a "heathen Chinee," as to mention the subject of preserving the pulps of teeth. They never heard of such a thing. They know arseneous acid destroys teeth, and this accomplishment is their high mission and ambition. Little or no excavations of the cavity follows when it is filled, and in a short time afterwards the patient returns to the mourners bench where vile sinner is again punished by extermination. Two fees have been collected, and the patient now departs, with *exalted* conceptions of the dental art. Much of the work done by the better American operators is that of taking out and doing over what these unholy hands every day demolish. Nor does the mischief stop at their operations in this branch. In artificial teeth, the caricatures are still worse and more of them. At the doorways of their offices the specimens of dentures brought from Paris, and their own "begettings," are enough to astonish the most incredulous. The mere sight of them is enough to make a patient weep, as he passes up to the "bull ring of these slaughter pens."

But what must the ordeal be when a patient is compelled to wear one of these products? If the missionary field is in need of laborers, here in Brazil is the commencement of a mighty work.

Of the character and predisposition to caries of the teeth in Brazil, I may say no country can excel it. The teeth are darker color, and a turgid condition of gums quite common. The tooth substance possesses a brightness and a sensitiveness, unusual in the States. There appears much destitution of lime salts—a starved condition of the teeth, insomuch that little or nothing can be done, excepting to remove them. Taints of syphilas prevail to no small extent throughout the Empire. Add to these the depressing influence of climate, humid and hot, with diet farinaceous and fruits, and the vital economy is slow and sluggish to respond to therapeutic agents. Very little certainty can be placed on remedial agents. Medicines in much larger doses are required in all pathological conditions. Their action is so feeble that the experienced practitioner would be surprised at the difference of power. Hence to be guided right with promises of success, require some exercise of caution and prudence.

A number of Brazilians in dental practice, have organized a society for the objects usual to such bodies. I have a copy of the by-laws which govern them, placed in my hands, and a translation made. As a curiosity, or one quite differently governed from American, I shall present it to some of our dental journals for publication. It will repay perusal.

